# Prioritizing Key Resilience Indicators to Support Coral Reef Management in a Changing Climate

**DOI:** 10.1371/journal.pone.0042884

**Published:** 2012-08-29

**Authors:** Tim R. McClanahan, Simon D. Donner, Jeffrey A. Maynard, M. Aaron MacNeil, Nicholas A. J. Graham, Joseph Maina, Andrew C. Baker, Jahson B. Alemu I., Maria Beger, Stuart J. Campbell, Emily S. Darling, C. Mark Eakin, Scott F. Heron, Stacy D. Jupiter, Carolyn J. Lundquist, Elizabeth McLeod, Peter J. Mumby, Michelle J. Paddack, Elizabeth R. Selig, Robert van Woesik

**Affiliations:** 1 Marine Programs, Wildlife Conservation Society, Bronx, New York, United States of America; 2 Department of Geography, University of British Columbia, Vancouver, British Columbia, Canada; 3 Centre de Recherches Insulaires et Observatoire de l'Environnement, Centre National de la Recherche Scientifique-Ecole Pratique des Hautes Etudes Papetoai, Moorea, Polynesie Francaise; 4 Australian Institute of Marine Science, Townsville, Queensland, Australia; 5 Australian Research Council Centre of Excellence for Coral Reef Studies, James Cook University, Townsville, Queensland, Australia; 6 Department of Biological Sciences, Macquarie University, Sydney, New South Wales, Australia; 7 Division of Marine Biology and Fisheries, Rosenstiel School of Marine and Atmospheric Science, University of Miami, Miami, Florida, United States of America; 8 Institute of Marine Affairs, Chaguaramas, Trinidad and Tobago, West Indies; 9 School of Biological Sciences, The University of Queensland, Brisbane, Queensland, Australia; 10 Indonesian Program, Wildlife Conservation Society, Bogor, Java, Indonesia; 11 Department of Biological Sciences, Simon Fraser University, Burnaby, British Columbia, Canada; 12 Coral Reef Watch, National Oceanic and Atmospheric Administration, Silver Spring, Maryland, United States of America; 13 Coral Reef Watch, National Oceanic and Atmospheric Administration, Townsville, Queensland, Australia; 14 Marine Geophysical Laboratory, School of Engineering and Physical Sciences, James Cook University, Townsville, Queensland, Australia; 15 Fiji Country Program, Wildlife Conservation Society, Suva, Fiji; 16 National Institute of Water & Atmospheric Research, Hamilton, New Zealand; 17 The Nature Conservancy, Honolulu, Hawaii, United States of America; 18 Marine Spatial Ecology Lab, School of Biological Sciences, University of Queensland, St Lucia, Queensland, Australia; 19 Department of Biological Sciences, Santa Barbara City College, Santa Barbara, California, United States of America; 20 Science+Knowledge Division, Conservation International, Arlington, VA, United States of America; 21 Department of Biological Sciences, Florida Institute of Technology, Melbourne, Florida, United States of America; Swansea University, United Kingdom

## Abstract

Managing coral reefs for resilience to climate change is a popular concept but has been difficult to implement because the empirical scientific evidence has either not been evaluated or is sometimes unsupportive of theory, which leads to uncertainty when considering methods and identifying priority reefs. We asked experts and reviewed the scientific literature for guidance on the multiple physical and biological factors that affect the ability of coral reefs to resist and recover from climate disturbance. Eleven key factors to inform decisions based on scaling scientific evidence and the achievability of quantifying the factors were identified. Factors important to resistance and recovery, which are important components of resilience, were not strongly related, and should be assessed independently. The abundance of resistant (heat-tolerant) coral species and past temperature variability were perceived to provide the greatest resistance to climate change, while coral recruitment rates, and macroalgae abundance were most influential in the recovery process. Based on the 11 key factors, we tested an evidence-based framework for climate change resilience in an Indonesian marine protected area. The results suggest our evidence-weighted framework improved upon existing un-weighted methods in terms of characterizing resilience and distinguishing priority sites. The evaluation supports the concept that, despite high ecological complexity, relatively few strong variables can be important in influencing ecosystem dynamics. This is the first rigorous assessment of factors promoting coral reef resilience based on their perceived importance, empirical evidence, and feasibility of measurement. There were few differences between scientists' perceptions of factor importance and the scientific evidence found in journal publications but more before and after impact studies will be required to fully test the validity of all the factors. The methods here will increase the feasibility and defensibility of including key resilience metrics in evaluations of coral reefs, as well as reduce costs. Adaptation, marine protected areas, priority setting, resistance, recovery.

## Introduction

Coral reefs are undergoing a major ecological disruption associated with climate change and human impacts that may forewarn changes among less sensitive ecosystems [1]. Will coral reefs persist through climate change and under what conditions? Can local management be used to increase their resilience? The coral reef science community is frequently asking such questions and they are likely to apply to other climate-impacted ecosystems (Table 1). The response to these questions has been mixed. Some researchers suggest more basic research is needed to understand resilience drivers, while others extrapolate from experimental manipulations or model forecasts to propose specific actions to help support reef resilience [2], [3], [4], [5]. Despite the urgency and apparent utility of these recommendations, field observations of important aspects of resilience, such as recovery, suggest that empirical observations are frequently inconsistent with purported theory [6]. This prompts the need for a current and full evaluation of factors deemed critical to supporting resilience.

**Table 1:** Questions and answers addressed in this study.

**Perceived importance of resilience factors**		
1.	Q:	What are the most important factors influencing coral reef resistance/recovery/resilience?
	A:	Of the 60+ factors considered there are only 11 that pass the test of expert and peer-reviewed literature consensus.
2.	Q:	How are the factors of resistance/recovery related?
	A:	They are not strongly related, which indicates that they can be evaluated and used to identify sites separately.
3.	Q:	If they are negatively correlated (i.e. represent trade offs), which factors still support resilience?
	A:	They are not. Therefore, each can be used independently.
4.	Q:	Which factors are positively correlated with resilience and should these be the key factors used to identify priority sites for management?
	A:	They are not. Therefore, each can be used independently.
**Sociology of resilience factors**		
5.	Q:	Do scientists uniformly share views on resistance/recovery/resilience or are there academic, experience, or cognitive cliques, clusters or camps?
	A:	Variation was random among the scientist's responses and, therefore, there was no evidence for cliques.
6.	Q:	Which factors share the most and least agreement among scientists?
	A:	The study scales these factors to suggest priorities for future research based on the variance in consensus.
**Empirical evidence, literature review, and prioritizing research**		
7.	Q:	What is the scientific evidence in support of factors considered to be the most important factors influencing resistance/recovery/resilience?
	A:	The evidence at the experimental and modeling level is only strong for a few of the eleven factors and this finding clearly identifies future research needs in this discipline.
8.	Q:	Which factors are considered most important but weakly supported by scientific evidence?
	A:	The influence of currents and light, reef connectivity, coral growth, size distributions, herbivore diversity and rates of reef erosion and complexity.
9.	Q:	What are the current priorities for research?
	A:	Evaluating the above factors are among the key priorities.
**Informing management decision-making**		
10.	Q:	Can the factors be defensibly scaled and is this scaling useful for prioritizing sites for management?
	A:	Yes, they can be scaled by evidence and expert consensus and this scaling greatly improves identifying and prioritizing sites based on resilience criteria.
11.	Q:	Would excluding some factors increase the robustness and defensibility of a resilience assessment?
	A:	Yes, including a large number of variables with little know relationship to resilience weakens and increases the cost of the resilience assessment approach. The evaluation developed in this paper will increase the defensibility of resilience evaluations.

Key questions examined in the study and their answers.

Ecological resilience can be defined as the capacity of an ecosystem to absorb recurrent disturbances or shocks and adapt to change while retaining essentially the same function and structure [7], [8]. Two key components of resilience are resistance, the ability of an ecological community to resist or survive a disturbance, and recovery, the rate a community takes to return to its original condition [9], [10]. Although resilience includes much more complexity than this, such as non-linear (threshold) dynamics and reinforcing feedbacks, the concepts of resistance and recovery are thought to be both tangible and important for management [11] and are therefore the focus of this assessment.

Despite gaps between resilience theory and field observations, the rapid rate of climate change disturbance has elevated demand for immediate solutions and management intervention among coral reef ecosystems [12], [13]. For instance, ocean warming is already impacting and reorganizing coral reef ecology on a large scale in the Western Indian Ocean [14]. Efforts are underway to inform conservation and management through identification of sites with high resistance to change and recovery from disturbance [15], [16]. For example, the IUCN has developed a protocol for assessing coral reef resilience in this way to define management priorities [16]. Although such site selection processes are crucial to the spatial management of coral reef resilience, empirical criteria to support these decisions are few. It is therefore critical that the scientific community develops resilience selection criteria based on the current state of knowledge and identifies key research priorities for future study.

By examining coral reef responses to disturbance across a range of past oceanographic and management conditions, site selection criteria for coral reef resilience can be developed that reflect how known disturbances and local environmental conditions have shaped present reef communities [13], [17]. Relevant conditions may include a range of physical factors such as reef hydrographic conditions and connectivity [6], [18], [19]; biological factors such as coral diversity, disease, and herbivory [20]; and habitat factors such as nutrients inputs, habitat complexity, and human impacts [4], [21]. However, having a wide range of physical and biological factors alone is not sufficient to develop sound resilience selection criteria. Factors must also be supported by science with substantial empirical evidence, weighted by the strength of the evidence linking factors to resistance and recovery.

Here we develop empirical selection criteria for prioritizing coral reef management and conservation in the face of climate change. These criteria are intended to identify reefs with the greatest resilience to climate disturbance so that local managers can support the persistence of local coral reef ecosystems. We also identify key research priorities for coral reef resilience, based on levels of perceived importance and areas of debate within the coral reef scientific community.

## Results and Discussion

### Perceived importance and empirical evidence of resilience factors

Among reef experts there was general agreement on combined resilience scores among factors (Fig. S1), but there was little overlap between the lists of top-ten ranked factors for the perceived importance of resistance and that for recovery, showing that these processes are thought to represent distinct components of reef resilience (Table 2, Table 3). Resistance factors perceived to be most important included the presence of stress-resistant coral species, which are less susceptible to thermally driven mortality [22]; the presence of stress-resistant symbionts, which are less vulnerable to heat stress [23]; and the presence of high annual temperature variability on a given reef, which can promote coral tolerance to anomalous temperatures [24].

**Table 2:** Scaled importance of resilience factors.

	Perceived importance (0 to 10)			Scientific evidence (−5 to +5)			Feasibility (0 to 10)
Ecological factor	Resilience	Resistance	Recovery	Resilience	Resistance	Recovery	
**(1) Resistant coral species**	**15.57**	**8.70**	**6.87**	**7.15**	**4.07**	**3.07**	**8.04**
**(2) Temperature variability**	**13.96**	**8.14**	5.82	**6.14**	**3.64**	2.50	**7.71**
Stress-resistant symbionts	**13.39**	**7.75**	5.64	**5.36**	**3.36**	2.00	3.19
**(3)Nutrients(pollution)**	**13.25**	**6.04**	**7.21**	**5.59**	**2.44**	**3.15**	5.63
**(4) Sedimentation**	**12.63**	**5.59**	**7.04**	**4.78**	**2.20**	**2.58**	6.73
**(5) Coral diversity**	**12.43**	**6.04**	6.39	4.11	**2.04**	2.07	**7.07**
**(6) Herbivore biomass**	**11.75**	4.29	**7.46**	**4.96**	1.64	**3.32**	**7.44**
**(7) Physical human impacts**	**11.67**	4.89	6.78	**4.81**	**1.96**	**2.85**	6.38
**(8) Coral disease**	**11.59**	**6.06**	5.54	3.81	**2.31**	1.50	6.43
Tidal mixing	**11.58**	**6.46**	5.13	4.41	**2.50**	1.91	4.83
**(9) Macroalgae**	11.46	3.89	**7.57**	**4.70**	1.33	**3.37**	**8.48**
**(10) Recruitment**	11.43	3.46	**7.96**	**4.89**	1.04	**3.86**	6.67
**(11) Fishing pressure**	11.39	4.32	7.07	**4.43**	1.46	**2.96**	**7.04**
Herbivore diversity	11.00	4.36	6.64	4.00	1.54	2.46	**7.33**
Habitat complexity	10.64	**5.08**	5.56	2.81	1.29	1.52	6.04
Connectivity	10.61	3.04	**7.57**	3.13	0.61	2.52	2.70
Mature colonies	10.39	4.21	6.18	2.81	1.07	1.74	**7.07**
Light (stress)	10.27	**6.31**	3.96	3.15	**2.31**	0.84	6.04
Coral size class distribution	10.08	4.81	5.27	2.58	1.19	1.38	6.88
Substrate suitability	10.00	2.39	**7.61**	2.93	0.36	**2.57**	6.52
Upwelling	9.83	5.04	4.78	2.63	1.46	1.17	4.71
Coral growth rate	9.79	2.71	**7.07**	1.79	−0.46	2.26	4.37
Proximity of other coastal habitats	9.67	4.04	5.63	3.39	1.36	2.04	**7.14**
Hard coral cover	9.50	3.71	5.79	3.14	0.88	2.27	**8.82**
Rapidly growing species	9.36	2.64	**6.71**	2.14	−0.64	**2.79**	6.89
Topographic complexity	9.19	4.74	4.44	2.26	1.22	1.04	6.19
Physical impacts	9.16	4.04	5.12	3.24	1.31	1.93	6.82
Wind mixing	8.00	4.00	4.00	2.71	1.52	1.19	4.45
Crustose coralline algae	7.81	2.54	5.27	0.35	0.00	0.35	6.62
Bioerosion rate	7.54	3.29	4.25	2.07	0.82	1.25	4.57
Exotics and invasives	7.00	3.04	3.96	2.42	0.92	1.50	5.00

Summary of the scaled perceived importance, scientific evidence, and feasibility of measurement for the top 31 factors. Perceived importance and feasibility are based on responses from 28 coral reef experts. Scientific evidence is based on a review of the journal literature with a distinct objective scale based on the level of evidence (see SI methods). Resilience scores are the sum of resistance and recovery scores. Values in bold indicate the top 10 values in each column; the 11 ecological factor names in bold indicate the feasible (feasibility>5) ecological factors which ranked among the top ten factors for perceived importance or empirical evidence of resilience.

**Table 3:** Estimated parameters for the bivariate resilience relationships.

	Response	Covariate	Intercept	Slope	Pearson Correlation
(a)	*PI Resistance score*	*PI Recovery score*	-	-	0.08
(b)	*EE Resistance score*	*EE Recovery score*	-	-	0.09
(c)	Resistance PI	Resistance EE	1.88 [1.44, 2.32]	1.59 [1.38, 1.80]	0.94
(d)	Recovery PI	Recovery EE	3.40 [2.69, 4.10]	1.24 [0.93, 1.55]	0.83
(e)	SD Resistance PI	Mean Resistance PI	3.49 [2.99, 3.98]	−0.23 [−0.34, −0.14]	−0.67
(f)	SD Recovery PI	Mean Recovery PI	3.06 [2.39, 3.75]	−0.15 [−0.26, −0.04]	−0.47
(g)	SD Resistance EE	Mean Resistance EE	-	-	−0.12
(h)	SD Recovery EE	Mean Recovery EE	-	-	−0.29

Model estimates for 31 factors based on the responses of 28 coral reef scientists. Relationships are: between resistance and recovery for (a) their perceived importance (PI) and (b) the scientific empirical evidence (EE); between perceived importance and scientific evidence for (c) resistance and (d) recovery; and between the mean and standard deviation of respondent scores for (e) resistance, (f) recovery, (g) empirical evidence, and (h) recovery. Values are median estimates and 95% uncertainty intervals (in parentheses); models are presented only for relationships with a clear linear trend (i.e. uncertainty intervals for slope parameter not spanning zero). Estimates include Pearson correlation coefficients, as the assignment of response and covariate was arbitrary. Intercept and slope values were not given if relationships were not statistically significant.

The most important factors for recovery included high levels of coral recruitment to replenish denuded locations [25]; suitable substrate for coral settlement and survival [26]; and low cover of macroalgae, which in high abundance can directly kill corals, trap sediment, prevent coral settlement, and dominate benthic space [27], [28]. Together, the top-ranked factors show that the most resilient reefs are expected to be those with high fish and coral diversity [29]; and few human impacts [30]. The ten highest-ranked factors for perceived importance also showed considerable overlap (70%) with those having the highest empirical evidence, demonstrating that the factors scientists perceive to be important are generally supported by published literature. As was the case for perceived importance, the factors showing evidence for resistance differed substantively from those with evidence for recovery (Table 3).

Identifying the top-ten ranked factors for resilience independently for perceived importance and scientific evidence, showed some overlap in factors, but produced a total list of 13 factors (Table 2). From this list, we only included factors that were considered feasible to assess (average feasibility scores >5), which resulted in a final list of 11 key factors for resilience management and conservation, ranging from the presence of stress-resistant corals to areas of reduced fishing pressure. Using only this final list of 11 key factors, we developed a site-selection framework for management.

To calculate resilience scores for a given reef, each of the 11 key factors was given a 5-point Likert scale value (0-none; 5-highest possible) to quantify its level of function and then weighted by its evidence score. These weighted factors were then averaged at each location to provide a single resilience score that could be readily compared among reefs (Text S1). The framework therefore represents a feasible approach that is based on the best available science for identifying the reefs most likely to persist through climate change.

### Informing coral reef management for resilience to climate change

Two approaches have been applied to set management and conservation priorities for supporting the natural resilience of coral reefs: measure as many variables as possible and select sites with the best set of positive characteristics; or measure a feasible set of factors with scientific support for promoting resilience. While the former approach has been applied recently [16], we believe the latter approach will lead to greater adoption and success in supporting coral reef resilience because it adopts a reduced set of factors that are both manageable and defensible, and therefore more likely to be implemented.

**Figure 1:**
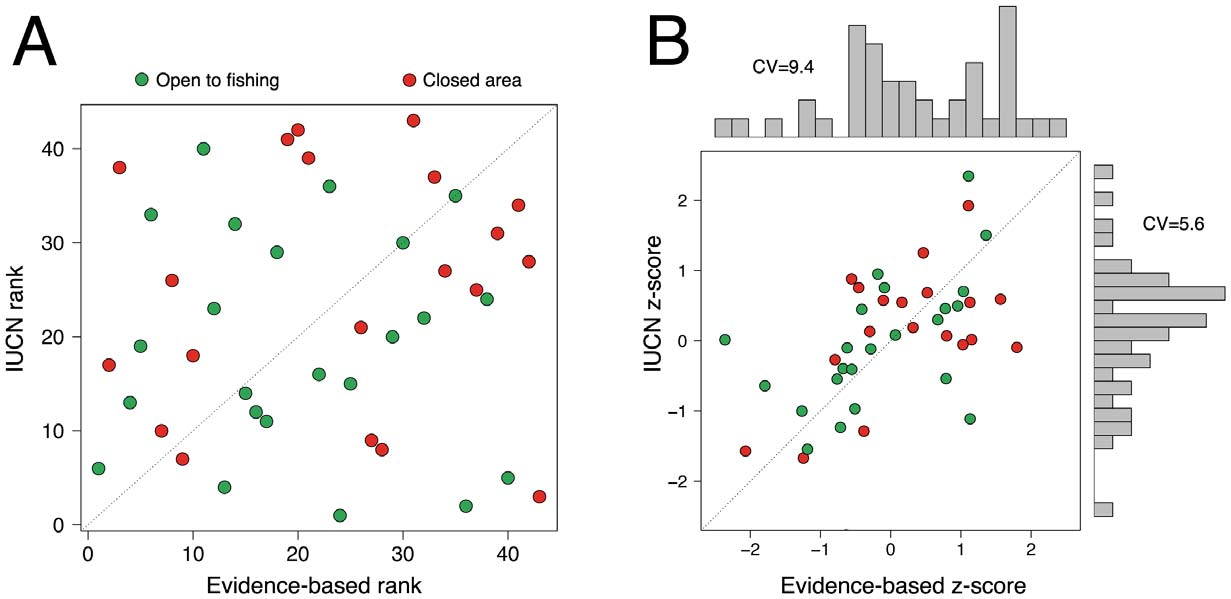
Relationship between IUCN and evidence-based rankings for sites in Karimunjawa, Indonesia. (A) Scatterplot of the relationship between IUCN and evidence-based rankings for the field evaluation of fished (green) and protected (red) coral reef sites in Karimunjawa. IUCN scores are based on 61 unweighted factors while evidence-based rankings are based on 11 weighted factors. (B) Scatterplot of the relationship between standardized (score minus mean-score divided by two times score standard deviation (SD)) IUCN and evidence-based score. Score coefficients of variation (CV; SD/mean*100) are provided alongside plot marginal histograms to illustrate central tendencies.

We compared our evidence-based rankings to rankings produced when using the 61-factor IUCN resilience assessment protocol [16] for the multi-use Karimunjawa Marine Park, in central Java, Indonesia (Text S1, Fig. S2). We compared the site-ranking scores produced by each framework to determine whether they produced similar results and discern which scheme provided the clearest differentiation among site-resilience scores. The resilience rankings among sites (n = 43) differed substantially, with little correlation between them (Cor = 0.07) indicating they represent divergent views about which reefs would most likely persist as the climate changes (Table S3, Fig. 1a). In addition, the 61 factor IUCN ranking system had a higher central tendency than our evidence-based framework (Fig. 1b) showing that the IUCN scores tended to regress toward their group average, as might be expected by the inclusion of a high number of potentially indiscriminant factors.

While intuitively appealing, including large numbers of factors in a resilience assessment may be both impractical and ineffective. The inclusion of more factors in a given framework lowers the importance of each factor in the end result and serves to make surveys more resource intensive and costly, decreasing the likelihood they can or will be used in practice. Consequently, optimal resilience assessments should be tailored to a sufficient and demonstrated set of factors that will maximize the efficiency and utility of the approach. It is in this way that our evidence-based framework makes a major advance toward an optimal resilience assessment framework for coral reefs. Periodic updates that integrate new research across experts and evidence will serve to refine this approach in future. Further, once these factors are used to identify potentially resilient reefs, monitoring change over time will allow additional tests of the value of these factors in influencing reef resistance and recovery. The consensus arising from this study is that, while there are potentially many factors involved in coral reef ecosystem resilience, there are actually only a few for which there is evidence of strong effects. This builds on the developing consensus in the broader ecological literature that ecosystems are complex but frequently controlled by just a few strong variables operating at a given scale [31], [32].

### Research priorities for coral reef resilience

Future resilience assessments that reflect scientific understanding about coral reefs will involve new and unexplored areas of coral reef ecology. While some potentially important areas, like genetic rates of adaptation, lie beyond the immediate scientific horizon, our scoring scheme provides insight into factors that constitute current research priorities. To estimate this for resistance, recovery, and resilience, we calculated a research priority score individually for each factor. This score is based on the ratio of importance to evidence scores among the 31 baseline factors determined as important in the workshop and plotted against a scientific consensus score, based on the average coefficient of variation (CV) for perceived importance and empirical evidence from among survey respondents (Text S1). This method effectively plots those factors thought to be important but with low empirical evidence against the level of controversy within the sampled scientific community. It should be noted that our assessment does not identify interactions among the factors, which may alter their importance.

**Figure 2:**
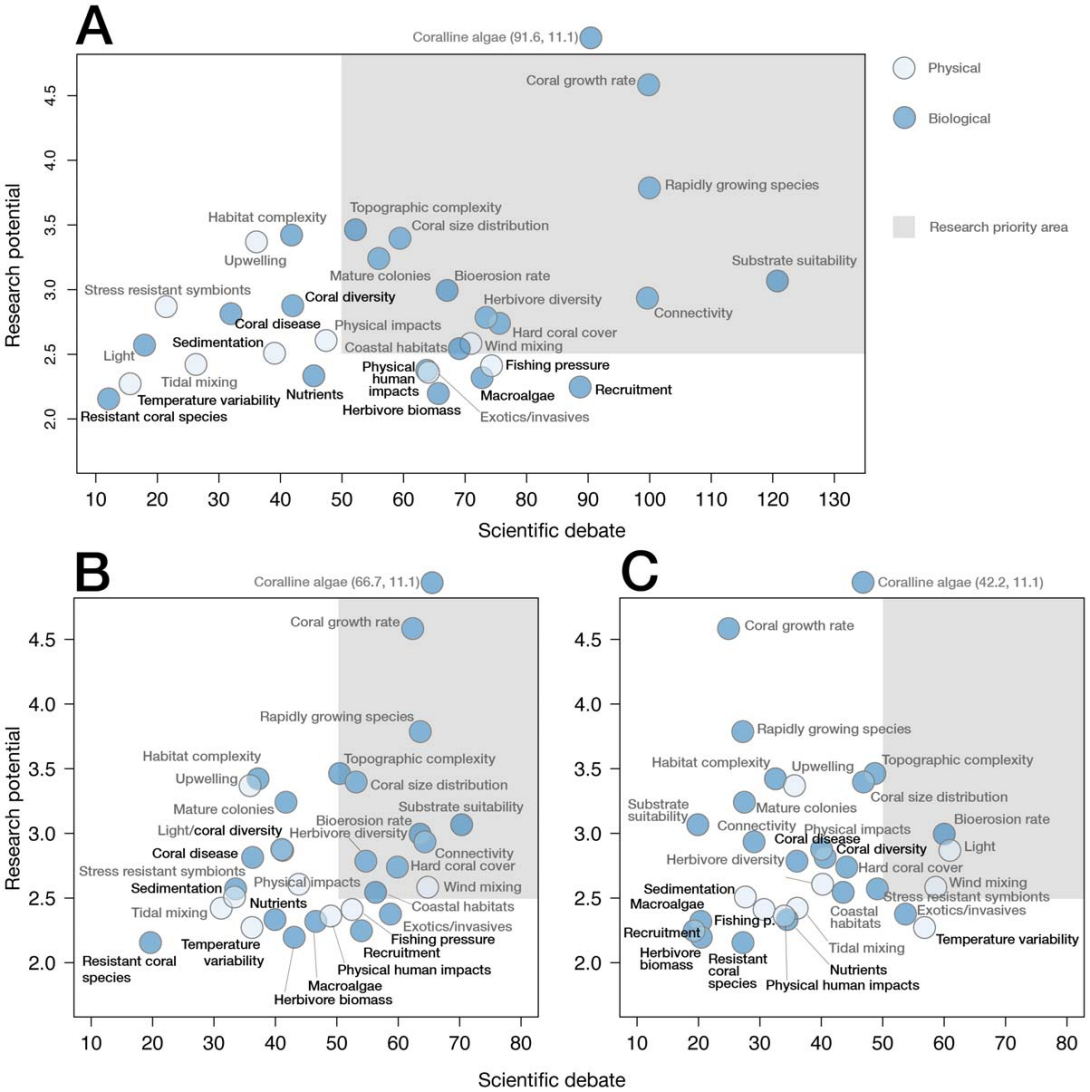
Relationship between scientific consensus and research potential. Scientific consensus (expert opinion coefficient of variation) vs. the research potential (importance/evidence ratio) for the 31 factors for the resilience for (A) resilience, based on the sum of resistance and recovery scores; (B) recovery, and (C) resistance. Y-axis values are means for each factor based on expert scores (n = 28).

Plotting research potential against scientific consensus revealed three key areas for future coral reef resilience research that can be partitioned into physical and biological processes (Fig. 2). The physical factors that require future research attention include weather-driven water mixing and the effects of light penetration. The biological factors included connectivity, coral growth and size distributions, herbivore diversity, rates of bioerosion, substrate suitability and the importance of topographic complexity (Tables S1 and S2 provide descriptions of each of these variables and relevant literature). Importantly, none of the 11 key factors were among the priority research areas. Although the 11 key factors need to be the focus of further applied research for management purposes, additional research should be directed towards the less-established factors identified by this group and the IUCN methodology which may have as yet unrecognized importance.

## Methods

### Ethics

Ethics clearance was not necessary because only the opinions of the researchers involved in the workshop were canvassed. All researchers were aware that their responses were being used for research and individual responses were anonymized. Additional details are provided in Text S1.

To develop a list of factors relevant to supporting coral reef resilience, we brought together approximately 50 coral reef scientists to address 11 key questions concerning the resilience of coral reefs (Table 1; Text S1). Participants were asked to evaluate 61 potential resilience factors currently used by the International Union for the Conservation of Nature coral reef assessment group [16]. From this, the participants reduced the 61 factors down to 31, based on experience and discussions (Tables S1, S2). Post-workshop, 28 coral reef scientists independently scored the 31 factors based on their perceived importance from personal experience and again based on the empirical evidence from scientific studies in terms of the factors ability to promote resistance to thermal stress and in promoting recovery from any type of disturbance. Respondents were also asked to rate the feasibility of measuring or assessing each factor. The factors were then modeled using Bayesian intercept-only models of scores to estimate the mean response and the variation among respondents in terms of their scientific understanding (Text S1). We then evaluated the group responses against the existing scientific literature to evaluate and scale the evidence for the original 11 key reef resilience questions (Tables S1, S2).

## Supporting Information

Text S1 **Methods** **and Results.**
(DOC)

Figure S1 **Multi-dimensional scaling of the responses to the (a) 31 and (b) top 8 factors evaluated for perceived effects of the factors on coral reef resilience.**
(TIF)

Figure S2 **Map of Karimunjawa Islands and associated coral reefs and the 43 sites studied for resilience to climate change disturbances.** Sites were split evenly into three groups based on the 11 key evidence-based factors and colored green for high, yellow for medium, and red for low climate resilience. Values next to sites are the rankings based on the 11 key evidence-based factors, with the unweighted and full 61 IUCN criteria in parentheses. Closed circles are no-take areas and triangles are general use zones.
(TIF)

Table S1 **Empirical evidence for factors relating to resistance and the evidence score (−5 to +5) based on evaluations from 28 coral reef experts.**
(DOC)

Table S2 **Empirical evidence for factors relating to recovery and the evidence score (−5 to +5) based on evaluations from 28 coral reef experts.**
(DOC)

Table S3 **Pair-wise matrix of the Pearson product correlation coefficients for comparisons of the resilience rankings produced for the study sites in Karimunjawa.** Scores for individual factors were not scaled in the method highlighted in the first (our study of 31 factors) and 5^th^ [16] columns and rows. Scaling for the others is continuous, based on the perceived importance of 31 selected most important variables and scaling the 11 variables based on the scientific evidence. Details are described in Text S1.
(DOC)
