# Supplementary material for: Prioritizing Key Resilience Indicators to Support Coral Reef Management in a Changing Climate
Source: PLoS One. 2012 Aug 29;7(8):e42884. doi: 10.1371/journal.pone.0042884 (PMC3430673; doi:10.1371/journal.pone.0042884)
Supplement: Text S1 — Methods and Results. (DOC) [file pone.0042884.s001.doc]

**Text S1. Methods and Results**

This supplementary material describes the methods to evaluate resilience factors, a sociological analysis of expert response data, a description of the application of the results to an evaluation of a seascape in Indonesia, and statistical analyses of the responses and the formulation of the metrics employed in the manuscript.

**Evaluation of resilience factors**

Based on an objective evaluation of the scientific literature described below, only two of 31 factors, resilient taxa and temperature variability, had experimental evidence of conferring resistance (Table S1). In addition, two factors, coral growth and abundance of rapidly growing species, were negatively related for resistance but not for recovery, scoring them low for evidence of conferring resilience (Table S2, Table 2). Multiple lines of evidence as being important for resistance found support for only the presence of resistant species. Nevertheless, this is not likely to be a key factor used to identify resilient sites because it may indicate high levels of past disturbance as much as future resilience . Evidence for recovery was generally scored lower than evidence for resistance, with only recruitment, macroalgae, nutrient pollution, and resistant species scored as having experimental evidence for influence on recovery. Expert opinion and evidence from journal publications were closely and positively correlated for the 31 factors, with a 0.94 correlation coefficient for resistance and 0.83 for recovery (Table S3).

Scientific evidence was evaluated based on -5 to +5 scale ranging from all evidence contrary (-5) to all evidence supportive (+5) for the resistance or recovery response. Evidence for most factors when largely limited to descriptive and observation-based studies was scaled to +1 if supportive and -1 if not. If findings were repeated and positive correlations consistently found than +2 was given, if natural or manipulative experimental evidence was available than +3 given, if modeling studies were supportive than +4 was given, and when supported by all lines of evidence and a broad consensus of scientists than +5 was given. Correlation is frequently the basis for exploring cause and effect relationship through experimentation and modeling and often, after developing inductive hypotheses, the beginning point for deductive investigation. While correlation is not causation it does suggest a relationship worthy of investigation and here we suggest it is a good point for a minimum level of evidence to include in an evaluation based on existing science.

This study demonstrates the need for more experimental analysis of individual factors before being more certain in any site evaluations. The dearth of experimental or BACI (before-after-control-impact) evidence for many factors may reflect biases that limit funding for large-scale and risky science and favor the evaluation of small-scale and tractable factors. As such for example, the existence of evidence for temperature variability and stress resistant symbionts may be due to the ease and appeal of experimental tests of those resilience factors.

**Sociology of resilience research**

Multidimensional scaling was employed to test whether the respondents were clustered in distinct expert groups, based on their scoring of the ecological factors. A matrix of correlation coefficients (28 x 28) was developed from least-squared regression of the response scores for the ecological factors between the responses of each of the 28 respondents. Correlation coefficients were computed using PI_resilience_ (the sum of PI_recovery_ and PI_resistance_) scores for all 31 indicators, and again for the top 15, the top 10 and top eight ranked indicators from Table 1. The function *mdscale*in Matlab 7.12 were then employed to construct scalar product matrices that reproduce the original correlation matrices, and could be represented in two dimensions.

Mapping these first two Eigenvalues from the scalar product matrices produced from the correlation matrix of responses to all 31 ecological factors (Fig S1a), does not suggest any distinct clustering of the respondents. Possible clustering becomes more evident based on correlation matrices produced from scores of only the top ranked indicators. The representation of the correlation matrix of responses to the top eight ranked factors, suggest the possibility of one large cluster of respondents (top right quadrant) and an overall ordering of other respondents (Fig S1b).

In all cases, however, the stress values, computed from difference between derived correlations (or distances) between respondents and original correlations were high (>0.2), indicating that the relative similarities between the respondents cannot be very adequately represented in two dimensional space. Further analyses yielded high stress values with less than four dimensions. This suggests no evidence for clustering of respondents into distinct expert groups.

The investigators listed their years in the profession from 2 to 30 and there was no significant relationship between either the mean or variances in their scores and the age of the profession. Consequently, there is no evidence for either shared or clustered views or an age effect in the responses to resilience factors.

**Evidence-based framework**

To characterize survey results among reef scientists we estimated resistance and recovery values using a set of Bayesian linear models for perceived importance, scientific evidence and feasibility. To handle any potential correlation between resistance and recovery estimates , we employed a bivariate normal model given each factor:

$P(y_{1ji},y_{2ji})\sim BvN((u_{1j},u_{2j}),\Sigma)$ (1)

where *y_1ji_* and *y_2ji_* denote resistance and recovery scores for factor *j* for respondent *i* respectively. Priors for factor means, μ, were the Normal sample means and precisions for each factor individually and Σ was Inverse-Wishart(I_n_, 3) for each factor. Note that while respondent scores were integer values, this modeling step yielded continuous estimates of the average score for each factor. For feasibility we simply estimated the factor-level estimate, using a Normal intercept-only model:

$y_{ji}\sim N(u_{j},\sigma_{j})$ (2).

Because not every respondent answered every question 4.9% of all factors were not scored, leading to missing data for a small subset of factors; however these few missing values were readily estimated from within the Bayesian model framework as additional parameters. All models were run using the PyMC package for the Python programming language . Models were run for 100,000 iterations with a 90,000 step burn-in period. All models were examined for evidence of autocorrelation and convergence in parameter chains, as well as goodness of fit.

Using the highest posterior density (HPD) estimates from the Bayesian models, we selected the top ten-ranked resistance and recovery factors that were positive for either perceived importance (>5) or scientific evidence (>0) and examined them for feasibility. Eleven of the top-ranked factors were deemed feasible (feasibility HPD estimates >5), providing an evidence-based set of factors from which to conduct resilience assessments.

**Applying resilience assessments**

In 2009, a resilience assessment of 43 coral reef sites in and around the multi-zoned Karimunjawa Marine National Park (central Java) was completed using the IUCN (2009) methodology, whereby 61 ecological factors were measured or semi-quantitatively assessed and scaled on a five-point score for resilience (Fig. S2). To test whether the rankings changed if resilience scores were based on the 11 evidence-based resilience scores, the sites were re-analyzed here using the weights for evidence (Table S1, S2).

The resultant rankings for the sites based on the two methods were not correlated (Table S3), highlighting different prioritizations. For example, seven sites were not identified as a priority for high resilience and increased protection by the IUCN 61 factors method and there is less spread among the sites without weighting leading to less confidence in site selection by ecological criteria (Fig 1b). The analyses also suggests there are a number of sites with high resilience that are not currently protected and inclusion in no-take areas would increase their chances for persistence. Park zones are assessed every 3 to 5 years by park management and resilience criteria are among the information used in their decision-making. Using only factors for which there is good scientific evidence produces different and more scientifically robust results and is expected to produce more defensible management decision-making when these assessments are undertaken.

**Priorities for research and management**

Two metrics were developed for estimating the relative priorities for research among the 31 ecological factors. The first is the degree of scientific debate:

*Scientific debate = [cv(PI_resilience_) + cv(ESE_resilience_)]/2*

The cv (PI_resilience_) and the cv (ESE_resilience_) is the coefficient of variation of the respondents’ scores for perceived importance (PI) and empirical scientific evidence (ESE), respectively, of an ecological factor for resilience (the sum of the scores recovery and resistance).

This metric evaluates the lack of agreement among study respondents; a high score indicates low agreement. The factors for which the mean empirical scientific evidence score for resistance or recovery was zero or negative had to be omitted from the scientific consensus analysis (coral growth rates, rapidly growing species and crustose coralline algae, see Table 3). The negative score indicates that the scientific evidence does not support the hypothesis that an ecological factor is related to increased resistance or recovery.

The second metric is the research potential:

*Research Potential = PI_resilience_ / (ESE_resilience_*+5*)*

This metric is a measure of the disparity between the perceived importance of a factor for resilience and the empirical scientific evidence for the hypothesis that the factor confers resilience. The empirical scientific evidence score is re-scaled to 0-10 to facilitate comparison.

A third metric was developed to rank the relative priorities for management, based on the perceived importance of a factor and the feasibility for measurement:

*Management Priority = PI_resilience_ × (Feasibility/10)*

This metric scales the perceived importance for resilience of an ecological factor by the feasibility of measuring and evaluating the factors. Since both the perceived importance and the feasibility are on scales of 0 to 10, the maximum possible score for management priority is 10. This metric is one of many ways to estimate the priorities for management, particularly relevant in cases where resources may be limited. Table 3 displays the rank of each factor according to the three metrics, as well as the raw values of the metrics for each factor.

**References**
