# Supplementary figures and images for: Prioritizing Key Resilience Indicators to Support Coral Reef Management in a Changing Climate
Source: PLoS One. 2012 Aug 29;7(8):e42884. doi: 10.1371/journal.pone.0042884 (PMC3430673; doi:10.1371/journal.pone.0042884)

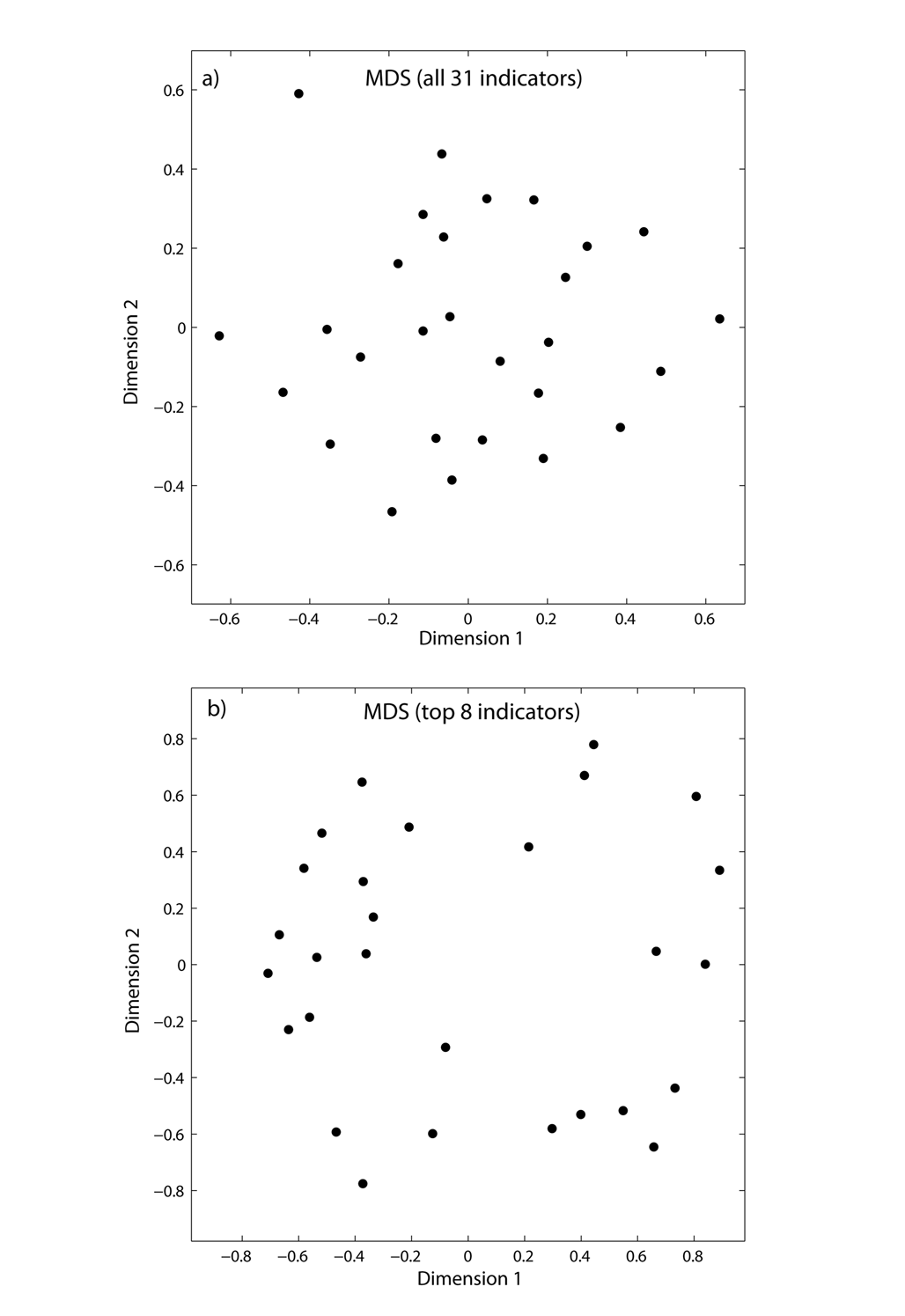

Supplement: Figure S1 — Multi-dimensional scaling of the responses to the (a) 31 and (b) top 8 factors evaluated for perceived effects of the factors on coral reef resilience. (TIF) [file pone.0042884.s002.tif]

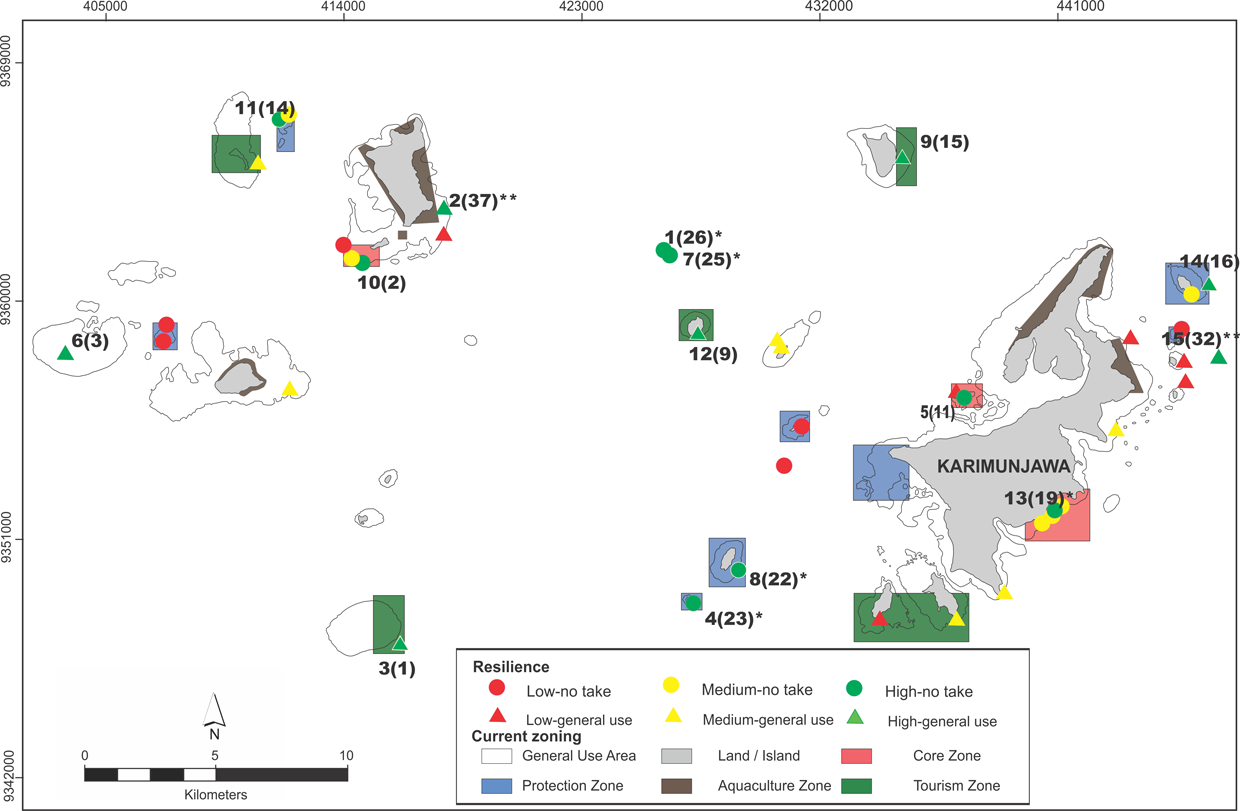

Supplement: Figure S2 — Map of Karimunjawa Islands and associated coral reefs and the 43 sites studied for resilience to climate change disturbances. Sites were split evenly into three groups based on the 11 key evidence-based factors and colored green for high, yellow for medium, and red for low climate resilience. Values next to sites are the rankings based on the 11 key evidence-based factors, with the unweighted and full 61 IUCN criteria in parentheses. Closed circles are no-take areas and triangles are general use zones. (TIF) [file pone.0042884.s003.tif]
