# Supplementary material for: Prioritizing Key Resilience Indicators to Support Coral Reef Management in a Changing Climate
Source: PLoS One. 2012 Aug 29;7(8):e42884. doi: 10.1371/journal.pone.0042884 (PMC3430673; doi:10.1371/journal.pone.0042884)
Supplement: Table S1 — Empirical evidence for factors relating to resistance and the evidence score (−5 to +5) based on evaluations from 28 coral reef experts. (DOC) [file pone.0042884.s004.doc]

Table S1. Empirical evidence for factors relating to resistance and the evidence score (-5 to +5) based on evaluations from 28 coral reef experts.

| **Empirical scientific evidence for resistance** | **Statement of evidence** | **Key citations** |
| --- | --- | --- |
| Resistant species | Resistant species (e.g. massive corals) are often not impacted by disturbance and a high abundance of resistant species, by definition, confers resistance |  |
| Temperature variability | Temperature variability, or the previous exposure of corals to different thermal regimes, has been demonstrated to increase resistance to bleaching in both field observations and experimental manipulations. |  |
| Stress-resistant symbionts | There is clear field and experimental evidence that different symbionttaxa lead to variation in bleaching tolerance both within and among coral species. Moreover, reefs in high temperature environments also tend to be dominated by corals with heat tolerant symbionts. |  |
| Reduced light stress | Higher solar radiation acts synergistically with high temperatures to increase bleaching susceptibility. Reduced solar radiation from cloud cover or physical structures that provide shade (e.g. emergent rocks or other corals) are thought to increase resistance to bleaching. |  |
| Water mixing (not weather) | Regular mixing from multiple oceanographic factors, primarily persistent oceanic currents, waves, or tidal currents, can provide cooling that protects reefs from thermal stress. This is especially true of upwelling, oceanic waves and tidal currents. Seasonally variable, but regular events such as monsoons can prevent growth of warm anomalies but can be disturbed by climate patterns such as ENSO. |  |
| Coral disease | Few studies have directly tested how disease affects bleaching sensitivity. Instead, research has focused on the effect of temperature on pathogen virulence, how disease outbreaks follow bleaching episodes (suggesting corals are more susceptible), and how disease might become more common as climate change continues. |  |
| Nutrient pollution | Field and experimental evidence suggests that nutrient pollution can reduce coral reef resistance to stress, but differences have been observed based on coral species, morphology, type of nutrient, level of nutrients and local context. |  |
| Coral diversity | Coral diversity may increase resistance, but this likely depends on the species composition and their species-specific sensitivities or tolerances to disturbance. Overall, the association between diversity and resistance remains unclear. |  |
| Sedimentation | The effects of increased sediments on corals, widely studied in both classical recent literatures are linked to resistance properties of corals. In synergy with SST, increased sediment and nutrients have been shown to decrease the thermal tolerance of corals causing bleaching during marginal increase in SST. |  |
| Anthropogenic physical impacts | Several studies have illustrated that there is a strong negative relationship between anthropogenic physical impacts (especially reef trampling and/or diving, ship groundings and coral mining/dredging) to coral reefs and their ability to resist stressors. Physical destruction may not kill coral colonies entirely, but even partial mortality and weakening increases susceptibility to thermally induced coral bleaching, disease outbreak or and reduce the reproductive potential of individuals. However, the degree of resistance exhibited by coral reefs or colonies may be dependent on the scale and frequency of the disturbance. |  |
| Habitat complexity | There is little evidence for habitat complexity per se conferring resistance. More complex habitats may have physical factors that enhance water movement and flush toxins. |  |
| Upwelling | Upwelling can reduce thermal stress during bleaching episodes and buffer stress through higher heterotrophy. However, upwelling does not always occur at the time of year when temperature anomalies occur. A potential negative impact is the advection water with low aragonite saturation. |  |
| Topographic complexity | Small-scale topographic complexity on a reef results in self-shading, cooling or shading that can reduce light stress during the long still hot periods that often precede the high temperatures that can cause bleaching. More resistance is conferred upon sites that are topographically complex |  |
| Size class distribution | There is little scientific evidence that the evenness across coral-colony size classes increases resistance to environmental stress. Yet, colony mortality is less frequent for large colonies than for small colonies and, within a given coral species, small encrusting juvenile colonies show more resistance to thermal stress than mature colonies. Moreover, the presence of large colonies on a reef shows that they have resisted stress events in the past |  |
| Fishing pressure | The ability to definitively link fishing pressure and resistance is difficult, due to the indirect impact of fishing pressure on corals and problems quantifying fishing pressure. |  |
| Herbivore diversity | Functional diversity within the herbivore guild may enhance coral resistance but there is little direct evidence for this. |  |
| Mature colonies | Low evidence rating and high relative variance indicate a weak indicator, with opposing evidence depending on the type of disturbance. During storms, mature larger colonies are mechanically stronger or more likely to remain as remnants, which provide a basis for recovery. Bleaching causes a reduction of mean colony size, which supports the notion that larger colonies have higher mortality or are broken up into smaller colonies, indicating a negative relationship. |  |
| Proximity of other habitats | Low evidence and high variance in the rating indicate a debatable indicator that depends on how other habitats are defined. Where other habitats are seagrass and mangroves, they prevent sediment from reaching a reef, thus reducing external stressors that smother corals, thus enhancing resistance. Coastal proximity and thus higher turbidity, however could protect corals from direct radiation. |  |
| Herbivore biomass | No clear evidence the herbivory increases resistance. It is possible that reduced algal competition might help corals withstand other stressors but no clear evidence. |  |
| Physical impacts | Storms/high wave energy may actually be beneficial in reducing coral bleaching. Majority of studies did not specifically address the effects of storm damage on coral bleaching resistance and recovery, other than commenting generally about the potentially additive stress of storms but hurricanes can reduce water temperatures and reduce thermal stress. |  |
| Water mixing (weather) | Weather events on several scales can reduce temperatures or change light reaching corals, helping corals resist thermal stress. However, weather is unpredictable at climate change scales, preventing an analysis of the patterns of change and potential protection they may bring. Even if cyclones increase in some way, their impact on reefs is largely driven by storm tracks that are unpredictable beyond a few days. |  |
| Macroalgae | The impact of macroalgae on resistance is not clear though potential factors are generally negative. Factors can work to counteract one another. For example, macroalgae can reduce growth rates, shade can reduce bleaching, and disease transmission from algae can divert coral resources. |  |
| Recruitment | Mixed evidence surrounds the thermal sensitivity of coral recruits and small size classes, compared to larger corals, with some evidence suggesting small corals bleach more severely, while a great number of studies suggest coral recruits and small size classes are more resistant to bleaching and mortality. |  |
| Coral cover | Perceived to be weakly important but reviews of coral cover and resistance to disturbance usually show no correlation. This most likely due to two factors: high coral cover often includes a higher proportion of more susceptible taxa, and sites with high coral cover often suffer a larger percentage loss with disturbance. |  |
| Bioerosion | There is little evidence linking bioerosion to resistance to disturbances but it could reduce the strength of the carbonate framework. |  |
| Population explosions, exotics and invasive species | There is some evidence that coral predators, such as *Acanthasterplanci*, may cause preferential mortality of thermally sensitive corals, leading to survivorship and dominance of thermally resistant taxa. Some ecological theory suggests that higher diversity communities may be more resistant to invasion (therefore presence of invasive species may indicate a compromised community), but this is contested and poorly tested on coral reefs. |  |
| Connectivity | There is no real basis for connectivity enhancing resistant, other than the potential supply of resistant taxa. However, effectively quantifying connectivity, is challenging |  |
| Rapidly growing species | Trade-off theory suggests physiological tradeoffs between rapid growth and resistance to environmental stress. Resistance and growth rate are often inversely related. For example, corals with a capacity to rapidly grow are more susceptible to environmental stress, whereas corals that do not have the capacity to rapidly grow are generally more resistant to environmental stress. |  |
| Coral growth rates | There is some indirect evidence through lack of visual stress banding that corals with higher long-term mean extension rates coupled with lower levels of chronic stress may have resisted past bleaching events, however this has not yet been confirmed with paired field observations of individual corals. As coral growth rates exhibit complex responses to local stressors direct measurements of local stressors would likely be more predictive. |  |
| Crustose coralline algae | There is little evidence linking CCA with resistance to disturbance of corals. |  |
| Substrate suitability | There is no evidence that substrate suitability enhances resistance, though it is correlated with other perceived resistance factors, including the presence of grazers and low macroalgal density. |  |

**References**

1. Loya Y, Sakai K, Yamazato K, Nakano Y, Sambali H, et al. (2001) Coral bleaching: the winners and the losers. Ecology Letters 4: 122-131.

2. Riegl Bl (2002) Effects of the 1996 and 1998 positive sea-surface temperature anomalies on corals, coral diseases and fish in the Arabian Gulf (Dubai, UAE). Marine Biology 140: 29-40.

3. Golbuu Y, Victor S, Penland L, Idip D, Emaurois C, et al. (2007) Palau's coral reefs show differential habitat recovery following the 1998-bleaching event. Coral Reefs 26: 319-332.

4. Foster KA, Foster G, Tourenq C, Shuriqi MK (2011) Shifts in coral community structures following cyclone and red tide disturbances within the Gulf of Oman (United Arab Emirates). Marine Biology: 1-14.

5. Donner SD, Kirata T, Vieux C (2010) Recovery from the 2004 coral bleaching event in the Gilbert Islands, Kiribati. Atoll Research Bulletin: 1-25.

6. McClanahan TR (2007) Response of the coral reef benthos and herbivory to fishery closure management and the 1998 ENSO disturbance. Oecologia 155: 169-177.

7. Brown BE, Downs CA, Dunne RP, Gibb SW (2002) Exploring the basis of thermotolerance in the reef coral *Goniastrea aspera*. Marine Ecology Progress Series 242: 119-129.

8. Oliver TA, Palumbi SR (2011) Do fluctuating temperature environments elevate coral thermal tolerance? Coral Reefs: 1-12.

9. Castillo KD, Helmuth BST (2005) Influence of thermal history on the response of Montastraea annularis to short-term temperature exposure. Marine Biology.

10. Thompson DM, van Woesik R (2009) Corals escape bleaching in regions that recently and historically experienced frequent thermal stress. Proceedings of the Royal Society B-Biological Sciences 276: 2893-2901.

11. Williams GJ, Knapp IS, Maragos JE, Davy SK (2010) Modeling patterns of coral bleaching at a remote Central Pacific atoll. Marine Pollution Bulletin 60: 1467-1476.

12. Teneva L, Karnauskas M, Logan C, Bianucci L, Currie J, et al. (2011) Predicting coral bleaching hotspots: the role of regional variability in thermal stress and potential adaptation rates. Coral Reefs: 1-12.

13. Donner SD (2011) An evaluation of the effect of recent temperature variability on the prediction of coral bleaching events. Ecological Applications 21: 1718-1730.

14. Rowan R, Knowlton N, Baker AC, Jara J (1997) Landscape ecology of algal symbiont communities explains variation in episodes of coral bleaching. Nature 388: 265-269.

15. Glynn PW, Maté JL, Baker AC, Calderón MO (2001) Coral bleaching and mortality in Panamá and Ecuador during the 1997-1998 El Niño-Southern Oscillation event: spatial/temporal patterns and comparisons with the 1982-1983 event. Bulletin of Marine Science 69: 79-109.

16. Baker AC, Starger CJ, McClanahan TR, Glynn PW (2004) Corals' adaptive response to climate change. Nature 430: 741.

17. Fabricius KE, Mieog JC, Colin PL, Idip D, Van Oppen MJH (2004) Identity and diversity of coral endosymbionts (zooxanthellae) from three Palauan reefs with contrasting bleaching, temperature and shading histories. 13: 2445-2458.

18. Rowan R (2004) Thermal adaptation in reef coral symbionts. Nature 430: 742.

19. Berkelmans R, van Oppen MJH (2006) The role of zooxanthellae in the thermal tolerance of corals: a 'nugget of hope' for coral reefs in an era of climate change. Proceedings of the Royal Society B-Biological Sciences 273: 2305-2312.

20. Jones AM, Berkelmans R, Van Oppen MJH, Mieog JC, Sinclair W (2008) A community change in the algal endosymbionts of a scleractinian coral following a natural bleaching event: field evidence of acclimatization. Proceedings of the Royal Society B-Biological Sciences 275: 1359-1365.

21. Coles SL, Jokiel PL (1978) Synergistic effects of temperature, salinity, and light on the hermatypic coral *Montipora verrucosa*. Marine Biology 49: 187-195.

22. Fitt WK, Brown BE, Warner ME, Dunne RP (2001) Coral bleaching: Interpretation of thermal tolerance limits and thermal thresholds in tropical corals. Coral Reefs 20: 51-65.

23. Lesser MP, Farrell JH (2004) Exposure to solar radiation increases damage to both host tissues and algal symbionts of corals during thermal stress. Coral Reefs 23: 367-377.

24. Brown BE, Dunne RP, Scoffin TP, Le Tissier MDA (1994) Solar damage in intertidal corals. Marine Ecology Progress Series 105: 219-230.

25. Mumby PJ, Chisholm JRM, Edwards AJ, Andrefouet S, Jaubert J (2001) Cloudy weather may have saved Society Island reef corals during the 1998 ENSO event. Marine Ecology-Progress Series 222: 209-216.

26. Brown BE, Dunne RP (2008) Solar radiation modulates bleaching and damage protection in a shallow water coral. Marine Ecology-Progress Series 362: 99-107.

27. Skirving W, Guinotte J (2001) The sea surface temperature story on the Great Barrier Reef during the coral bleaching event of 1998. In: Wolanski E, editor. Oceanographic processes of coral reefs: physical and biological links in the Great Barrier Reef. Boca Raton, FL: CRC Press. pp. 356.

28. Skirving WJ, Heron SF, Steinberg CR, McLean C, Parker BAA, et al. (2010) Determining Thermal Capacitance for Protected Area Network Design in Palau. Silver Spring, MD: NOAA Technical Memorandum CRCP 12, NOAA Coral Reef Conservation Program. 316 p.

29. Hoeke R, Brainard R, Moffitt R, Merrifield M, Skirving W (2006) The role of oceanographic conditions and reef morphology in the 2002 coral bleaching event in the Northwestern Hawaiian Islands. Atoll Research Bulletin 543: 489-503.

30. Glynn PW (1988) El Niño-Southern Oscillation 1982-1983: nearshore population, community, and ecosystem responses. Annual Review of Ecology and Systematics 19: 309-345.

31. Glynn PW, Ault JS (2000) A biogeographic analysis and review of the far eastern Pacific coral reef region. Coral Reefs 19: 1-23.

32. Harvell CD, Mitchell CE, Ward JR, Altizer S, Dobson AP, et al. (2002) Climate Warming and Disease Risks for Terrestrial and Marine Biota. Science 296: 2158-2162.

33. Lesser MP, Bythell JC, Gates RD, Johnstone RW, Hoegh-Guldberg O (2007) Are infectious diseases really killing corals? Alternative interpretations of the experimental and ecological data. Journal of Experimental Marine Biology and Ecology 346: 36-44.

34. Rosenberg E, Ben-Haim Y (2002) Microbial diseases of corals and global warming. Environmental Microbiology 4: 318-326.

35. Ben-Haim Y, Thompson FL, Thompson CC, Cnockaert MC, Hoste B, et al. (2003) Vibrio coralliilyticus sp nov., a temperature-dependent pathogen of the coral Pocillopora damicornis. International Journal of Systematic and Evolutionary Microbiology 53: 309-315.

36. Bruno JF, Selig ER, Casey KS, Page CA, Willis BL, et al. (2007) Thermal stress and coral cover as drivers of coral disease outbreaks. Plos Biology 5: 1220-1227.

37. Martínez JL, Baquero F (2002) Interactions among Strategies Associated with Bacterial Infection: Pathogenicity, Epidemicity, and Antibiotic Resistance. Clinical Microbiology Reviews 15: 647-679.

38. Nordemar I, Nystrom M, Dizon R (2003) Effects of elevated seawater temperature and nitrate enrichment on the branching coral Porites cylindrica in the absence of particulate food. Marine Biology 142: 669-677.

39. Fabricius KE (2005) Effects of terrestrial runoff on the ecology of corals and coral reefs: review and synthesis. Marine Pollution Bulletin 50: 125-146.

40. Fabricius KE (2006) Effects of irradiance, flow, and colony pigmentation on the temperature microenvironment around corals: implications for coral bleaching? Limnology and Oceanography 51: 30–37.

41. Wooldridge SA (2009) Water quality and coral bleaching thresholds: Formalising the linkage for the inshore reefs of the Great Barrier Reef, Australia. Marine Pollution Bulletin 58: 745-751.

42. Wooldridge SA, Done TJ (2009) Improved water quality can ameliorate effects of climate change on corals. Ecological Applications 19: 1492-1499.

43. McClanahan TR, Maina JM, Muthiga NA (2011) Associations between climate stress and coral reef diversity in the western Indian Ocean. Global Change Biology 17: 2023-2032.

44. Nyström M, Graham NAJ, Lokrantz J, Norström AV (2008) Capturing the cornerstones of coral reef resilience: linking theory to practice. Coral Reefs 27: 795-809.

45. Cote I, Darling E (2010) Rethinking ecosystem resilience in the face of climate change. PLoS Biology 8: e1000438.

46. Rogers CS (1983) Sub-lethal and lethal effects of sediments applied to common Caribbean reef corals in the field. Marine Pollution Bullettin 14: 378-382.

47. Rogers CS (1990) Responses of coral reefs and reef organisms to sedimentation. Marine Ecology Progress Series 62: 185-202.

48. Gilmour J (1999) Experimental investigation into the effects of suspended sediment non fertilisation, larval survival and settlement in a scleractinian coral. Marine Biology 135: 451-462.

49. Wolanski E, Richmond RH, McCook L (2004) A model of the effects of land-based, human activities on the health of coral reefs in the Great Barrier Reef and in Fouha Bay, Guam, Micronesia. Journal of Marine Systems 46: 133– 144.

50. Hawkins JP, Roberts CM (1992) Effects of recreational SCUBA diving on fore-reef slope communities of coral reefs. Biol Conserv 62: 171-178.

51. Zakai D, Chadwick-Furman NE (2002) Impacts of intensive recreational diving on reef corals at Eilat, northern Red Sea. Biological Conservation 105: 179-187.

52. Chabanet P, Adjeroud M, Andrefouet S, Bozec YM, Ferraris J, et al. (2005) Human-induced physical disturbances and their indicators on coral reef habitats: a multi-scale approach. Aquatic Living Resources 18: 215–230.

53. Fox HE, Mous P, Pet J, Muljadi A, Caldwell RL (2005) Experimental assessment of coral reef rehabilitation following blast fishing. Conserv Biol: 98-107.

54. West JM, Salm RV (2003) Resistance and Resilience to Coral Bleaching: Implications for Coral Reef Conservation and Management. Conserv Biol 17: 956-967.

55. Glynn PW, D'Croz LD (1990) Experimental evidence for high temperature stress as the cause of the El Nino-coincident coral mortality. Coral Reefs 8: 181-191.

56. Chollett I, Mumby PJ, Cortes J (2010) Upwelling areas do not guarantee refuge for coral reefs in a warming ocean. Marine Ecology Progress Series 416: 47-56.

57. Nakamura T, van Woesik R (2001) Water-flow rates and passive diffusion partially explain differential survival of corals during the 1998 bleaching event. Marine Ecology Progress Series 212: 301–304.

58. Bena C, van Woesik R (2004) The impact of two bleaching events on the survival of small coral colonies (Okinawa, Japan). Bulletin of Marine Science 75: 115-125.

59. Cameron AM, Endean R (1985) Do long-lived species structure coral reef ecosystems? In: Gabrie C, Harmelin M, editors. 5th Int Coral Reef Congr. Tahiti. pp. 211-215.

60. Andres NG, Rodenhouse NL (1993) Resilience of corals to hurricanes: a simulation model. Coral Reefs 12: 167-175.

61. Soong K (1993) Colony size as a species character in massive reef corals. Coral Reefs 12: 77-83.

62. Selig ER, Bruno JF (2010) A global analysis of the effectiveness of marine protected areas in preventing coral loss. PLoS ONE 5: e9278.

63. Hargreaves-Allen V, Mourato S, Milner-Gulland EJ (2011) A Global Evaluation of Coral Reef Management Performance: Are MPAs Producing Conservation and Socio-Economic Improvements? Environmental Management 47: 684–700.

64. Mumby PJ, Harborne AR, Hedley JD, Zychaluk K, Blackwell PG (2006) Revisiting the catastrophic die-off of the urchin *Diadema antillarum* on Caribbean coral reefs: Fresh insights on resilience from a simulation model. Ecological Modelling 196: 131-148.

65. Bellwood DR, Hughes TP, Folke C, Nyström M (2004) Confronting the coral reef crisis. Nature 429: 827-833.

66. Nyström M (2006) Redundancy and response diversity of functional Groups: Implications for the resilience of coral reefs. Ambio 35: 30-35.

67. Shenkar N, Fine M, Loya Y (2005) Size matters: bleaching dynamics of the coral Oculina patagonica. Marine Ecology-Progress Series 294: 181-188.

68. Madin JS, Connolly SR (2006) Ecological consequences of major hydrodynamic disturbances on coral reefs. Nature 444: 477-480.

69. McClanahan TR, Ateweberhan M, Omukoto J (2008) Long-term changes in coral colony size distributions on Kenyan reefs under different management regimes and across the 1998 bleaching event. Marine Biology 153: 755-768.

70. Nugues MM, Roberts CM (2003) Partial mortality in massive reef corals as an indicator of sediment stress on coral reefs. Marine Pollution Bulletin 46: 314-323.

71. Alongi DM, McKinnon AD (2005) The cycling and fate of terrestrially-derived sediments and nutrients in the coastal zone of the Great Barrier Reef shelf. Marine Pollution Bulletin 51: 239-252.

72. Anthony KRN (2006) Enhanced energy status of corals on coastal, high-turbidity reefs. Marine Ecology-Progress Series 319: 111-116.

73. Anthony KRN, Connolly SR, Hoegh-Guldberg O (2007) Bleaching, energetics, and coral mortality risk: Effects of temperature, light, and sediment regime. Limnology and Oceanography 52: 716-726.

74. Tanner JE (1995) Competition between Scleractinian Corals and Macroalgae: an Experimental Investigation of Coral Growth, Survival and Reproduction. Journal of Experimental Marine Biology and Ecology 190: 151-168.

75. Foster NL, Box SJ, Mumby PJ (2008) Competitive effects of macroalgae on the fecundity of the reef-building coral Montastraea annularis. Marine Ecology Progress Series 367: 143-152.

76. Connell JH, Hughes TP, Wallace CC (1997) A 30-year study of coral abundance, recruitment, and disturbance at several scales in space and time. Ecological Monographs 76: 461–488.

77. Gardner TA, Cote IM, Gill JA, Grant A, Watkinson AR (2005) Hurricanes and Caribbean coral reefs: impacts, recovery patterns, and role in long-term decline. Ecology 86: 174-184.

78. Manzello DP, Brandt M, Smith TB, Lirman D, Hendee JC, et al. (2007) Hurricanes benefit bleached corals. Proceedings of the National Academy of Sciences USA 104: 12035–12039.

79. Fabricius K, De'ath G, Puotinen M, Done T, Cooper T, et al. (2008) Disturbance gradients on inshore and offshore coral reefs caused by a severe tropical cyclone. Limnology and Oceanography 53: 690–704.

80. Obura DO (2005) Resilience and climate change lessons from coral reefs and bleaching in the Western Indian Ocean. Estuarine, Coastal and Shelf Science 63: 353-372.

81. Skirving WJ, Strong AE, Liu G, Arzayus F, Liu C, et al. (2006) Extreme events and perturbations of coastal ecosystems. In: Richardson LL, LeDrew EF, editors. Remote Sensing of Aquatic Coastal Ecosystem Processes: Springer. pp. 11-26.

82. Dunne, Brown (2001) The influence of solar radiation on bleaching of shallow water reef corals in the Andaman Sea, 1993-1998. Coral Reefs 20: 201-210.

83. Manzello D, Hendee JC, Ward D, Hillis-Starr Z (2006) An evaluation of environmental parameters coincident with the partial bleaching event in St Croix, US Virgin Islands. 709-717 p.

84. Grimsditch GD, Salm RV (2006) Coral reef resilience and resistance to bleaching. Gland, Switserland: The Nature Conservancy. 52 p.

85. Salm RV, Done TJ, McLeod E (2006) Marine protected area planning in a changing climate. In: Phinney JT, Hoegh-Guldberg O, Kleypas J, Skirving W, Strong A, editors. Coral Reefs and Climate Change: Science and Management. Washington, DC: American Geophysical Union

86. Mumby PJ, Harborne AR, Williams J, Kappel CV, Brumbaugh DR, et al. (2007) Trophic cascade facilitates coral recruitment in a marine reserve. Proceedings of the National Academy of Sciences of the United States of America 104: 8362-8367.

87. Harriott VJ (1985) Mortality-rates of scleractinian corals before and during a mass bleaching event. Marine Ecology-Progress Series 21: 81-88.

88. Mumby PJ (1999) Bleaching and hurricane disturbances to populations of coral recruits in Belize. Marine Ecology Progress Series 190: 27-35.

89. Darling ES, McClanahan TR, Côté IM (2010) Combined effects of two stressors on Kenyan coral reefs are additive or antagonistic, not synergistic. Conservation Letters 3.

90. Graham NAJ, McClanahan TR, MacNeil MA, Wilson SK, Polunin NVC, et al. (2008) Climate warming, marine protected areas and the ocean-scale integrity of coral reef ecosystems. PLoS ONE 3: e3039.

91. Hutchings PA (1986) Biological destruction of coral reefs Coral Reefs 4: 239-252

92. Reaka-Kudla ML, Feingold JS, Glynn W (1996) Experimental studies of rapid bioerosion of coral reefs in the Galfipagos Islands Coral Reefs 15: 101-107.

93. Carreiro-Silva M, McClanahan TR (2001) Echinoid bioerosion and herbivory on Kenyan coral reefs: the role of protection from fishing Journal of Experimental Marine Biology and Ecology 262: 133-153

94. Bellwood DR, Hoey AS, Choat JH (2003) Limited functional redundancy in high diversity systems: resilience and ecosystem function on coral reefs Ecology Letters 6: 281–285.

95. McClanahan TR, Maina J, Starger CJ, Herron-Perez P, Dusek E (2005) Detriments to post-bleaching recovery of corals. Coral Reefs 24: 230–246.

96. Levine JM, D'Antonio CM (1999) Elton revisited: A review of evidence linking diversity and invasibility. Oikos 87: 15-26.

97. Gates RD, Edmunds PJ (1999) The physiological mechanisms of acclimatization in tropical reef corals. American Zoological 39: 30-43.

98. McClanahan T, Polunin N, Done T (2002) Ecological states and the resilience of coral reefs. Conservation Ecology 6.

99. Carilli J, Norris R, Black B, Walsh S, McField M (2009) Local Stressors Reduce Coral Resilience to Bleaching. PLoS ONE 4: e6324.

100. Carilli J, Norris RD, Black BA, Walsh SM, McField M (2010) Century-scale records of coral growth rates indicate that local stressors reduce coral thermal tolerance threshold. Global Change Biology 16: 1247-1257.

101. Jokiel P, Rodgers KS, Kuffner IB, Andersson AJ, Cox EF, Mackenzie FT (2008) Ocean acidification and calcifying reef organisms: a mesocosm investigation. Coral Reefs 27: 473-483.
