# Supplementary material for: Prioritizing Key Resilience Indicators to Support Coral Reef Management in a Changing Climate
Source: PLoS One. 2012 Aug 29;7(8):e42884. doi: 10.1371/journal.pone.0042884 (PMC3430673; doi:10.1371/journal.pone.0042884)
Supplement: Table S2 — Empirical evidence for factors relating to recovery and the evidence score (−5 to +5) based on evaluations from 28 coral reef experts. (DOC) [file pone.0042884.s005.doc]

Table S2. Empirical evidence for factors relating to recovery and the evidence score (-5 to +5) based on evaluations from 28 coral reef experts.

| **Empirical scientific evidence for recovery** | **Statement of evidence** | **Key citations** |
| --- | --- | --- |
| Resistant species | Resistant species, such as massive corals that remain after a disturbance can continue to grow and reproduce to promote recovery, although these are often slow-growing species and coral recovery may depend more on the recolonization of fast-growing branching and plating species. |  |
| Temperature variability | Temperature variability is thought to be an important but how past temperature exposure affects their rate of recovery from thermal stress events is not well studied. Corals with thermally tolerant symbionts exhibit slower growth rates, potentially making them less able to recover and re-grow following bleaching events. |  |
| Stress-resistant symbionts | Bleached corals can recover with more thermotolerant symbionts at both the coral colony and community-level, implying that reefs that already contain a high abundance of these symbionts will also be the fastest to recover. However, Reefs that contain thermotolerant symbionts bleach less severely than corals that do not, and thus the recovery of the two groups is difficult to compare. Moreover, there is some evidence for tradeoffs among symbionts that might result in corals that are thermotolerant showing slower growth rates and hence lower recovery. |  |
| Reduced light stress | There is little evidence to suggest that reduced light stress may increase recovery. |  |
| Water mixing (not weather) | There is currently little evidence that recovery of corals is likely to be greater in areas with water mixing. | none |
| Coral disease | Little evidence that high levels of disease impede recovery from bleaching. However, disease outbreaks often follow episodes of mass bleaching, which would imply slower recovery as corals expend resources to combat infection. |  |
| Nutrient pollution | Nutrient pollution is associated with decreased recovery following disturbance but studies recognize the challenge of separating the effects of multiple stressors, such sedimentation, overfishing from pure nutrients. |  |
| Coral diversity | There is limited evidence that coral diversity promotes recovery following disturbance. |  |
| Sedimentation | There is scientific evidence that can sediments can limit the recovery of coral reefs. It has been shown that sediment can smother corals tissue, and limit coral larvae settlement impairing coral recovery. Additionally sediments can also inhibit recovery and growth of inshore reefs in deposition areas, and as a result can modify the zonation of coral reefs. |  |
| Anthropogenic physical impacts | There is mixed evidence on the impact of physical anthropogenic disturbances on coral reef recovery. Most studies have linked anthropogenic physical impacts to coral lower growth rates, lower reproductive potential, fewer coral recruits, lower and survivorship and increased disease incidence. Conversely, other studies have found that these impacts (e.g. trampling, displacement of coral boulders, anchor damage, ship groundings, blast fishing, nuclear blasts and snorkeling/diving damage) created new coral habitat available for colonization by corals and certain fish species post impact. |  |
| Habitat complexity | No clear evidence that habitat complexity promotes recovery |  |
| Upwelling | No clear evidence or obvious mechanism by which upwelling would enhance recovery. |  |
| Topographic complexity | No evidence for topographic complexity increasing recovery of corals but few studies have investigated this factor. |  |
| Size class distribution | There is scientific evidence that evenness across size classes increases recovery. An even distribution across size classes indicates a recovering community, whereas the under-representation of juvenile colonies suggests recruitment failure and a suppressed recovery rate. Moreover, the lack of large coral colonies strongly suggests that the community is in the early stages of recovery, or in the late stages of decline, whereby the environmental stress caused high partial mortality, and fragmentation. |  |
| Fishing pressure | Increased coral recruitment and growth have been demonstrated on some reefs protected from fishing whereas no evidence has been found in others. |  |
| Herbivore diversity | Experimental evidence indicates that the presence of a diverse guild and functional groups of herbivores can enhance coral recovery. |  |
| Mature colonies | The presence and survival of mature colonies provides more propagules for colonizing free space after a disturbance. Larger mature colonies are more likely to form remnants, which can be major contributors to recovery through regrowth. |  |
| Proximity of other habitats | Recovery is enhanced by the presence of herbivore nursery habitats such as mangroves and seagrass. Yet, coastal estuarine environments can negatively impact reefs through high sediment loads that reduce the settlement of recruits and bleaching at lower bleaching thresholds. |  |
| Herbivore biomass | Most studies have linked increased herbivory to reduced macroalgal cover and an increase in coral recruitment despite higher corallivory. One study has gone further and shown that increased herbivore biomass led to a reversal in the reef trajectory from one of coral decline to coral recovery. Relative importance of fish and urchins varies geographically and with fishing intensity. |  |
| Physical impacts | Empirical evidence suggests that physical impacts (waves and storms) can reduce coral reef recovery from disturbance, but recovery depends upon the scale and intensity of disturbance, and extent of coral and substrate damage. |  |
| Water mixing (weather) | There is currently little evidence that recovery of corals is likely to be greater in areas with water mixing. | none |
| Macroalgae | Macroalgae is a significant factor limiting the recovery of corals following disturbance by increasing competition for benthic substrate, allelopathy and by trapping sediment that smothers coral recruits. |  |
| Recruitment | High rates of successful coral recruitment and survival enhance coral recovery rates following disturbance. |  |
| Coral cover | Coral cover is linked to increased resilience and recovery but most field studies showing no correlation between coral cover pre- or post-disturbance with recovery rates. |  |
| Bioerosion | There is evidence that bio-erosion from mostly macro-bioeroders (sea urchins, parrotfishes, gastropods) can impair coral reef recovery. There is no much evidence however, in relation to other bioeroders. |  |
| Population explosions, exotics and invasive species | Explosions of corallivorescan severely reduce long-term recovery from multiple perturbations to coral reefs. |  |
| Connectivity | Connectivity is thought to promote recovery of corals through the supply of coral larvae from less impacted locations although a recent meta-analysis of coral reef recovery dynamics showing no relationship between distance between reefs and recovery. Connectivity to seagrass and mangroves has been found to enhance the diversity of organisms on reefs that may be responsible for recovery processes. |  |
| Rapidly growing species | The type of coral present and rate at which those coral colonies are able to grow will determine the rate at which the reef recovers but fast growing corals are often absent early after a disturbance. |  |
| Coral growth rates | Reduced growth rates are associated with reduced cover on some reefs but the interaction between environment and coral life histories produces variable responses. |  |
| Crustose coralline algae | The interactive effects of settlement induction, competition and increased predation make the influence unclear. |  |
| Substrate suitability | Substrate availability is crucial for recovery by proving a framework for settlement and growth. High Substrate availability can also be a sign of low larval retention or high natural disturbance rates. |  |

**References**

1. Riegl Bl (2002) Effects of the 1996 and 1998 positive sea-surface temperature anomalies on corals, coral diseases and fish in the Arabian Gulf (Dubai, UAE). Marine Biology 140: 29-40.

2. Riegl B, Purkis S (2009) Model of coral population response to accelerated bleaching and mass mortality in a changing climate. Ecological Modelling 220: 192-208.

3. Burt J, Bartholomew A, Usseglio P (2008) Recovery of corals a decade after a bleaching event in Dubai, United Arab Emirates. Marine Biology 154: 27-36.

4. Glynn PW, Maté JL, Baker AC, Calderón MO (2001) Coral bleaching and mortality in Panamá and Ecuador during the 1997-1998 El Niño-Southern Oscillation event: spatial/temporal patterns and comparisons with the 1982-1983 event. Bulletin of Marine Science 69: 79-109.

5. Little AF, Van Oppen MJH, Willis BL (2004) Flexibility in algal endosymbioses shapes growth in reef corals. Science 304: 1492-1494.

6. Jones A, Berkelmans R (2010) Potential Costs of Acclimatization to a Warmer Climate: Growth of a Reef Coral with Heat Tolerant vs. Sensitive Symbiont Types. PLoS ONE 5.

7. Berkelmans R, Willis BL (1999) Seasonal and local spatial patterns in the upper thermal limits of corals on the inshore central Great Barrier Reef. Coral Reefs 18: 219-228.

8. Baker AC, Starger CJ, McClanahan TR, Glynn PW (2004) Corals' adaptive response to climate change. Nature 430: 741.

9. Berkelmans R, van Oppen MJH (2006) The role of zooxanthellae in the thermal tolerance of corals: a 'nugget of hope' for coral reefs in an era of climate change. Proceedings of the Royal Society B-Biological Sciences 273: 2305-2312.

10. Jones AM, Berkelmans R, Van Oppen MJH, Mieog JC, Sinclair W (2008) A community change in the algal endosymbionts of a scleractinian coral following a natural bleaching event: field evidence of acclimatization. Proceedings of the Royal Society B-Biological Sciences 275: 1359-1365.

11. LaJeunesse TC, Smith RT, Finney J, Oxenford H (2009) Outbreak and persistence of opportunistic symbiotic dinoflagellates during the 2005 Caribbean mass coral 'bleaching' event. Proceedings of the Royal Society B-Biological Sciences 276: 4139-4148.

12. Bruno JF, Selig ER, Casey KS, Page CA, Willis BL, et al. (2007) Thermal stress and coral cover as drivers of coral disease outbreaks. Plos Biology 5: 1220-1227.

13. Connell JH, Hughes TP, Wallace CC (1997) A 30-year study of coral abundance, recruitment, and disturbance at several scales in space and time. Ecological Monographs 76: 461–488.

14. Hughes TP, Baird AH, Bellwood DR, Card M, Connolly SR, et al. (2003) Climate Change, Human Impacts, and the Resilience of Coral Reefs. Science 301: 929-933.

15. Fabricius KE (2005) Effects of terrestrial runoff on the ecology of corals and coral reefs: review and synthesis. Marine Pollution Bulletin 50: 125-146.

16. Carilli J, Norris R, Black B, Walsh S, McField M (2009) Local Stressors Reduce Coral Resilience to Bleaching. PLoS ONE 4: e6324.

17. Nyström M, Graham NAJ, Lokrantz J, Norström AV (2008) Capturing the cornerstones of coral reef resilience: linking theory to practice. Coral Reefs 27: 795-809.

18. Cote I, Darling E (2010) Rethinking ecosystem resilience in the face of climate change. PLoS Biology 8: e1000438.

19. Rogers CS (1990) Responses of coral reefs and reef organisms to sedimentation. Marine Ecology Progress Series 62: 185-202.

20. Crabbe MJC, Smith DJ (2005) Sediment impacts on growth rates of Acropora and Porites corals from fringing reefs of Sulawesi, Indonesia. Coral Reefs 24: 437–441.

21. Acevedo R, Morelock J, Olivieri RA (1989) Modification of coral reef zonation by terrigenous sediment stress. Palaios 4: 92-100.

22. McManus JW, Reyes Jr RB, Nañola Jr CL (1997) Effects of some destructive fishing methods on coral cover and potential rates of recovery. Environmental Management: 69-78.

23. Riegl B, Luke KE (1998) Ecological parameters of dynamited reefs in the northern Red Sea and their relevance to reef rehabilitation. Mar Pollut Bull 37: 488-498.

24. Ebersole JP (2001) Recovery of fish assemblages from ship groundings on coral reefs in the Florida keys National Marine Sanctuary. Bulletin of Marine Science 69: 655-671.

25. Rogers KS, Cox EF (2003) The effects of trampling on Hawaiian corals along a gradient of human use. Biological Conservation 112: 383-389.

26. Chabanet P, Adjeroud M, Andrefouet S, Bozec YM, Ferraris J, et al. (2005) Human-induced physical disturbances and their indicators on coral reef habitats: a multi-scale approach. Aquatic Living Resources 18: 215–230.

27. Fox HE, Mous P, Pet J, Muljadi A, Caldwell RL (2005) Experimental assessment of coral reef rehabilitation following blast fishing. Conserv Biol: 98-107.

28. Chollett I, Mumby PJ, Cortes J (2010) Upwelling areas do not guarantee refuge for coral reefs in a warming ocean. Marine Ecology Progress Series 416: 47-56.

29. Done TJ, DeVantier LM, Turak E, Fisk DA, Wakeford M, et al. (2010) Coral growth on three reefs: development of recovery benchmarks using a space for time approach. Coral Reefs 29: 815-833.

30. Aronson RB, Precht WF (2006) Conservation, precaution, and Caribbean reefs. Coral Reefs 25: 441-450.

31. Mumby PJ, Harborne AR, Williams J, Kappel CV, Brumbaugh DR, et al. (2007) Trophic cascade facilitates coral recruitment in a marine reserve. Proceedings of the National Academy of Sciences of the United States of America 104: 8362-8367.

32. McClanahan TR, Ateweberhan M, Omukoto J (2008) Long-term changes in coral colony size distributions on Kenyan reefs under different management regimes and across the 1998 bleaching event. Marine Biology 153: 755-768.

33. Mumby PJ, Harborne AR (2010) Marine reserves enhance the recovery of corals on Caribbean reefs. PLoS One 5: e8657.

34. O'Leary JK, Braga JC, Potts DC, McClanahan TR (2011) Indirect consequences of fishing: reduction of coralline algae suppresses coral recruitment. Coral Reefs in press.

35. Bellwood DR, Hughes TP, Folke C, Nyström M (2004) Confronting the coral reef crisis. Nature 429: 827-833.

36. Nyström M (2006) Redundancy and response diversity of functional Groups: Implications for the resilience of coral reefs. Ambio 35: 30-35.

37. Bellwood DR, Hughes TP, Hoey AS (2006) Sleeping functional group drives coral-reef recovery. Current Biology 16: 2434-2439.

38. Burkepile DE, Hay ME (2008) Herbivore species richness and feeding complementarity affect community structure and function on a coral reef. Proceedings of the National Academy of Sciences of the United States of America 105: 16201-16206.

39. Golbuu Y, Victor S, Penland L, Idip D, Emaurois C, et al. (2007) Palau's coral reefs show differential habitat recovery following the 1998-bleaching event. Coral Reefs 26: 319-332.

40. Mumby PJ, Edwards AJ, Arias-Gonzalez JE, Lindeman KC, Blackwell PG, et al. (2004) Mangroves enhance the biomass of coral reef fish communities in the Caribbean. Nature 427: 533-536.

41. Birrell CL, McCook LJ, Willis BL (2005) Effects of algal turfs and sediment on coral settlement. Marine Pollution Bulletin 51: 408-414.

42. Mumby PJ, Hastings A (2008) The impact of ecosystem connectivity on coral reef resilience. Journal of Applied Ecology 45: 854-862.

43. Unsworth RKF, De Leon PS, Garrard SL, Jompa J, Smith DJ, et al. (2008) High connectivity of Indo-Pacific seagrass fish assemblages with mangrove and coral reef habitats. Marine Ecology-Progress Series 353: 213-224.

44. Wooldridge SA, Done TJ (2009) Improved water quality can ameliorate effects of climate change on corals. Ecological Applications 19: 1492-1499.

45. Edmunds PJ, Carpenter RC (2001) Recovery of Diadema antillarum reduces macroalgal cover and increases abundance of juvenile corals on a Caribbean reef. Proceedings of the National Academy of Sciences of the United States of America 98: 5067-5071.

46. Hughes TP, Rodrigues MJ, Bellwood DR, Ceccarelli D, Hoegh-Guldberg O, et al. (2007) Phase Shifts, Herbivory, and the Resilience of Coral Reefs to Climate Change. Current Biology 17: 360-365.

47. Halford A, Cheal A, Ryan D, Williams D (2004) Resilience to large-scale disturbance in coral and fish assemblages on the Great Barrier Reef. Ecology 85: 1892–1905.

48. Gardner TA, Cote IM, Gill JA, Grant A, Watkinson AR (2005) Hurricanes and Caribbean coral reefs: impacts, recovery patterns, and role in long-term decline. Ecology 86: 174-184.

49. Fabricius K, De'ath G, Puotinen M, Done T, Cooper T, et al. (2008) Disturbance gradients on inshore and offshore coral reefs caused by a severe tropical cyclone. Limnology and Oceanography 53: 690–704.

50. Kuffner IB, Walters LJ, Becerro MA, Paul VJ, Ritson-Williams R, et al. (2006) Inhibition of coral recruitment by macroalgae and cyanobacteria. Marine Ecology Progress Series 323: 107-117.

51. Colgan MW (1987) Coral reef recovery on Guam (Micronesia) after catastrophic predation by Acanthaster planci. Ecology 68: 1592-1605.

52. Hughes TP, Baird AH, Dinsdale EA, Moltschaniwskyj NA, Pratchett MS, et al. (2000) Supply-side ecology works both ways: the link between benthic adults, fecundity, and larval recruits. Ecology 81: 2241-2249.

53. Cros A, McClanahan T (2003) Coral transplant damage under various management conditions in the Mombasa Marine National Park, Kenya. Western Indian Ocean Journal of Marine Science 2: 127-136.

54. McClanahan TR, Maina J, Starger CJ, Herron-Perez P, Dusek E (2005) Detriments to post-bleaching recovery of corals. Coral Reefs 24: 230–246.

55. Penin L, Michonneau F, Carroll A, Adjeroud M (2011) Effects of predators and grazers exclusion on early post-settlement coral mortality. Hydrobiologia 663: 259-264.

56. Brown-Saracino J, Peckol P, Curran HA, Robbart ML (2007) Spatial variation in sea urchins, fish predators, and bioerosion rates on coral reefs of Belize. Coral Reefs 26: 71–78.

57. Lourey MJ, Ryan DAJ, Miller IR (2000) Rates of decline and recovery of coral cover on reefs impacted by, recovering from and unaffected by crown-of-thorns starfish *Acanthaster planci*: a regional perspective of the Great Barrier Reef. Marine Ecology Progress Series 196: 179-186.

58. Jones GP, Almany GR, Russ GR, Sale PF, Steneck RS, et al. (2009) Larval retention and connectivity among populations of corals and reef fishes: history, advances and challenges. Coral Reefs 28: 307-325.

59. Graham NAJ, Nash KL, Kool JT (2011) Coral reef recovery dynamics in a changing world. Coral Reefs 30: 283-294.

60. McClanahan TR, Ateweberhan M, Muhando CA, Maina J, Mohammed MS (2007b) Effects of climate and seawater temperature variation on coral bleaching and mortality. Ecological Monographs 77: 503-525.

61. van Woesik R, Sakai K, Ganase A, Loya Y (2011) Revisiting the winners and losers a decade after coral bleaching. Marine Ecology Progress Series 434: 67-76.

62. Edwards AJ, Clark S, Zahir H, Rajasuriya A, Naseer A, et al. (2001) Coral Bleaching and Mortality on Artificial and Natural Reefs in Maldives in 1998, Sea Surface Temperature Anomalies and Initial Recovery. Marine Pollution Bulletin 42: 7-15.

63. Edinger EN, Limmon GV, Jompa J, Widjatmoko W, Heikoop JM, et al. (2000) Normal coral growth rates on dying reefs: are coral growth rates good indicators of reef health. Marine Pollution Bulletin 40: 404-405.

64. Heyward AJ, Negri AP (1999) Natural inducers for coral larval metamorphosis. Coral Reefs 18: 273-279.

65. Harrington L, Fabricius K, De'ath G, Negri A (2004) Recognition and selection of settlement substrata determine post-settlement survival in corals. Ecology 85: 3428-3437.

66. Wilson SK, Graham NAJ, Pratchett MS, Jones GP, Polunin NVC (2006) Multiple disturbances and the global degradation of coral reefs: are reef fishes at risk or resilient? Global Change Biology 12: 2220-2234.

67. Victor S (2008) Stability of reef framework and post settlement mortality as the structuring factor for recovery of Malakai Bay Reef, Palau, Micronesia: 25 years after a severe COTS outbreak. Estuarine, Coastal and Shelf Science 77: 175-180.
