# Supplementary material for: Prioritizing Key Resilience Indicators to Support Coral Reef Management in a Changing Climate
Source: PLoS One. 2012 Aug 29;7(8):e42884. doi: 10.1371/journal.pone.0042884 (PMC3430673; doi:10.1371/journal.pone.0042884)
Supplement: Table S3 — Pair-wise matrix of the Pearson product correlation coefficients for comparisons of the resilience rankings produced for the study sites in Karimunjawa. Scores for individual factors were not scaled in the method highlighted in the first (our study of 31 factors) and 5th [16] columns and rows. Scaling for the others is continuous, based on the perceived importance of 31 selected most important variables and scaling the 11 variables based on the scientific evidence. Details are described in Text S1. (DOC) [file pone.0042884.s006.doc]

Table S3. Pair-wise matrix of the Pearson product correlation coefficients for comparisons of the resilience rankings produced for the study sites in Karimunjawa.

|  | Top 31 factors | | Strongest 11 factors | | Full 61 factors |
| --- | --- | --- | --- | --- | --- |
|  | No scaling | Perceived Resilience* | Perceived Resilience** | Scientific Evidence | IUCN (2009) |
| No scaling | 1 |  |  |  |  |
| Resilience, 31* | 0.95 | 1 |  |  |  |
| Resilience, 11** | 0.53 | 0.68 | 1 |  |  |
| Evidence, 11** | 0.53 | 0.68 | 0.98 | 1 |  |
| IUCN (2009) | 0.28 | 0.28 | 0.32 | 0.29 | 1 |

Scores for individual factors were not scaled in the method highlighted in the first (our study of 31 factors) and 5^th^ (IUCN 2009) columns and rows. Scaling for the others is continuous, based on the perceived importance of 31 selected most important variables and scaling the 11 variables based on the scientific evidence. Details are described in the methods.
